# Halfway between 2D and Animal Models: Are 3D Cultures the Ideal Tool to Study Cancer-Microenvironment Interactions?

**DOI:** 10.3390/ijms19010181

**Published:** 2018-01-18

**Authors:** Jessica Hoarau-Véchot, Arash Rafii, Cyril Touboul, Jennifer Pasquier

**Affiliations:** 1 Stem Cell and Microenvironment Laboratory, Weill Cornell Medical College in Qatar, Qatar Foundation, Education City, Doha 24144, Qatar; jeh2036@qatar-med.cornell.edu (J.H.-V.); jat2021@qatar-med.cornell.edu (A.R.); 2 Department of Genetic Medicine, Weill Cornell Medical College, New York, NY 10065, USA; 3 UMR INSERM U965, Angiogenèse et Recherche Translationnelle, Hôpital Lariboisière, 49 bd de la Chapelle, 75010 Paris, France; cyril.touboul@gmail.com; 4 Service de Gynécologie-Obstétrique et Médecine de la Reproduction, Centre Hospitalier Intercommunal de Créteil, Faculté de Médecine de Créteil UPEC, Paris XII, 40 Avenue de Verdun, 94000 Créteil, France; 5 INSERM U955, Equipe 7, 94000 Créteil, France

**Keywords:** tumor microenvironment, 3D culture, 3D anchorage independent culture, 2D culture, tumor proliferation, tumor migration, chemoresistance

## Abstract

An area that has come to be of tremendous interest in tumor research in the last decade is the role of the microenvironment in the biology of neoplastic diseases. The tumor microenvironment (TME) comprises various cells that are collectively important for normal tissue homeostasis as well as tumor progression or regression. Seminal studies have demonstrated the role of the dialogue between cancer cells (at many sites) and the cellular component of the microenvironment in tumor progression, metastasis, and resistance to treatment. Using an appropriate system of microenvironment and tumor culture is the first step towards a better understanding of the complex interaction between cancer cells and their surroundings. Three-dimensional (3D) models have been widely described recently. However, while it is claimed that they can bridge the gap between in vitro and in vivo, it is sometimes hard to decipher their advantage or limitation compared to classical two-dimensional (2D) cultures, especially given the broad number of techniques used. We present here a comprehensive review of the different 3D methods developed recently, and, secondly, we discuss the pros and cons of 3D culture compared to 2D when studying interactions between cancer cells and their microenvironment.

## 1. Introduction

Despite the advances in treatment over the last decades, cancer remains a leading cause of death worldwide. Treating cancer has been challenging due to the complexity and heterogeneity of tumors, leading to resistance to chemotherapy. This complexity is partly due to the interaction between the tumor and its microenvironment [1,2]. The tumor microenvironment (TME) consists of different non-cancer cell types and their stroma, such as fibroblasts, immune cells (lymphocytes and macrophages), mesenchymal cells, and endothelial cells (EC), which all have a specific role in the physiology, structure, and function of the tumor [3]. The tumor and its microenvironment induce reciprocal changes in their phenotypes and functions that sustain the ongoing process of tumor development and spreading [4,5,6,7]. Studying interactions between cancer and the TME involves developing optimal surrogate platforms where the complex features of cancer cells, such as migration, proliferation, and chemoresistance, can be investigated. This has been proved to be quite challenging both in vitro and in vivo due to the difficult task to reproduce all the complex tumoral and non-tumoral cell interactions.

Most of the published data regarding known cell-based processes is derived from experiments performed in two-dimensional (2D) conditions where cells are grown on rigid materials such as polystyrene and glass. These conventional cell monolayer cultures, grown under simplified and unrealistic conditions, do not fully reflect the essential physiology of real tissues. They modify the tissue-specific architecture (forced polarity, flattened cell shape), mechanical/biochemical signals, and subsequent cell-to-cell communication [8]. Despite these drawbacks, 2D cultures remain very attractive for laboratory purposes because of their simplicity and low cost.

When one wants to confirm a phenomenon or mechanism observed in vitro, the usual and common approach is to use standard animal testing, usually referred to as animal models. However, there are many concerns regarding the discomfort or the pain of animals under certain experimental conditions. Many experimental animals have compromised immune systems and do not offer the same stroma-tumor interaction as humans, which prevents the efficient translation of novel research to clinical settings [9]. Obtaining concordance between animal models and clinical trials still remains challenging, with an average rate of concordant results that barely reaches 8% [10,11]. Therefore, switching from 2D cultures to three-dimensional (3D) cultures is motivated by the need to create cellular models that better captures the complexities of tumor biology.

The ideal 3D model would eliminate the differences related to species that are usually encountered, allowing drug testing directly on human models. Defining optimal 3D models that best mimic the specificity of the tumor microenvironment seems to be of growing interest for the scientific community. When the number of publications on 3D models in the 90s barely reached 10 per year, during the last ten years, the increase has been exponential, reaching almost 1000 publications in 2016 alone. This is due to the emergence of many different new techniques that are potentially of great value in the context of tumor-TME interaction studies.

Here, we provide an overview of different culture methods in 3D, and discuss their use, challenges, and differences compared to 2D cell cultures. The topics covered in this review include cancer proliferation and migration, as well as resistance to chemotherapy. Our goal is to provide a comprehensive review of the benefits and drawbacks of both 2D and 3D cultures in the expanding field of tumor-TME interactions.

## 2. Different Models of 3D Culture

Working in 3D involves formation of spheroids. Spheroids are aggregates that can either be grown in suspension, encapsulated, or grown on the top of a 3D matrix using different 3D methods; each one has its specific advantages and disadvantages [12]. 3D methods can be divided into the following categories: (i) hanging drop methods; (ii) non-adherent surface methods; (iii) suspension culture; (iv) scaffolds-based: hydrogel; (v) magnetic levitation and bio-printing; (vi) microfluidic methods.

### 2.1. Hanging Drop Methods

**Figure 1:**
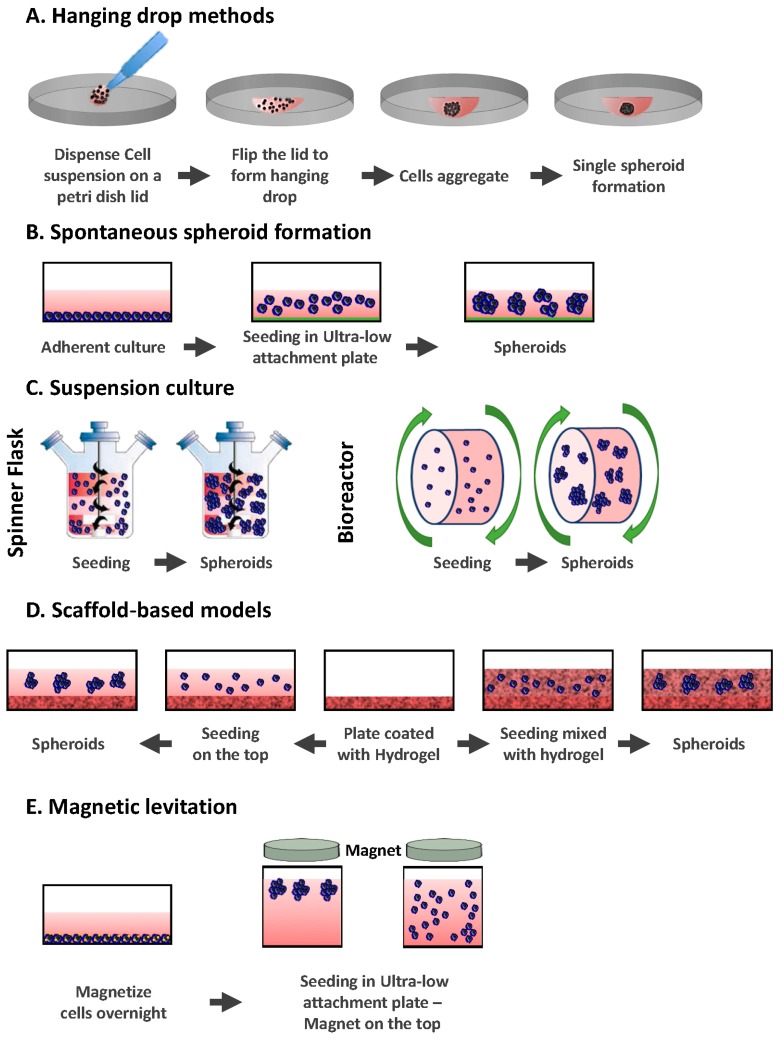
Common 3D techniques used for the creation of spheroids. (**A**) Hanging drop methods. Cells are deposited on a petri dish lid, which is flipped over a petri dish containing PBS; (**B**) ultra low attachment plates. Cells are seeded in an ultra-low attachment plate which prevents them from adhering; (**C**) suspension cultures. Cells are placed in spinner flasks (left) or bioreactors (right) and put under gravitational forces; (**D**) scaffold based-models. Cells are either seeded on the top of a hydrogel (left) or embedded in it (right); (**E**) magnetic levitation. Cells are magnetized in culture and attracted to a magnet located on the top.

The hanging drop method was originally a microbiology method used to study bacteria in a confined and controlled environment. In this technique, drops of cell suspensions are placed onto the underside of a petri dish lid (Figure 1A). The lid, where the cells hang due to surface tension, is then placed onto a petri dish, which contains PBS (phosphate buffer saline) to prevent dehydration of the droplets. The cells accumulate at the tip of the drop at the air-liquid interface, spontaneously aggregate, and finally form spheroids [13,14].

The hanging drop method does not require the use of any substance that can negatively affect the spheroids, as cells naturally adhere to each other and do not have to rely on matrices or scaffolds. However, drops with liquid volumes of more than 50 μL do not adhere to the petri dish (since the liquid surface tension is overcome by gravity) [15]. It can also be a challenge to change the medium without disturbing the spheroid.

Recently, hanging drop plates (HDP) have been used instead of petri dish plates, allowing the production of larger numbers of 3D spheroids per plate [16,17,18]. HDP have a bottom tray filled with a liquid that will keep a humid atmosphere inside the plate to prevent any evaporation/drying out. On the top of the tray sits a hanging drop plate with access holes where cell suspension samples can be placed to form hanging drops.

Overall, the hanging drop method is quite simple and consistent. It can produce one spheroid per drop for different cell lines [19]. Using HDPs is simpler compared to the original hanging drop method, as it can be manipulated by liquid handling robots nevertheless it remains quite expensive. HDP is compatible with high throughput screening and offers a new approach to testing a greater number of drugs on different cell lines while replicating a 3D in vivo biological context [16]. Indeed, the natural direct cell-cell and cell-ECM (Extra Cellular Matrix) interaction that takes place in-vivo is preserved. The main interest of this technique relies on the fact that it allows the creation of uniformly sized spheroids (in mono and co-culture). This was shown to be achievable from a small number of cells, which is a real advantage when working with rare patient-derived cells [20]. Spheroids can be maintained in culture for several weeks within the droplet array, allowing the development of complex experiments [17,18].

### 2.2. Spontaneous Spheroid Formation: Non-Adherent Surface Methods/Ultra Low Attachment Plates

Sutherland et al. pioneered techniques of spheroid production in 1970 [21,22]. They used a 3D in vitro system model to recreate the complexities of the multi-cellular tumor in order to study the response of human tumor cells to the effect of radiotherapy. In this method, ultra-low attachment plates are used by coating them with an inert substrate (agar or poly-2-hydroxyethyl methacrylate (poly-HEMA)), which prevents cells from attaching to the surface of the wells, forcing the cells to aggregate and form spheroids (Figure 1B) [19,23]. The method was improved by Ivasku and collaborators in 2006, using round and conical bottomed 96-well plates to efficiently produce 3D spheroids from cancerous and non-cancerous cells [24]. The plates used were coated with 0.5% poly-HEMA and dried for three days before adding the cells to them. Different cell lines were studied and when specific cell lines were not able to form spheroids, 2.5% of liquid reconstituted basement membrane was added to the suspensions. This led to the generation of compact 3D spheroids within 24 h after centrifugation.

It is also possible to use commercially pre-coated plates where the bottom surface is hydrophilic. The bottom surface is neutrally charged and covalently bound to a polystyrene vessel surface. This coating prevents adherence of the cells to the surface, instead forcing them to be in suspension and consequently form 3D spheroids. The coating is stable, non-cytotoxic, and non-degradable.

Spontaneous spheroid formation techniques are easy to use and allow a high throughput of spheroids (96 or 384 well plates). The method is relatively cheap when plates are coated in-house, but commercially pre-coated plates can be more expensive. Spheroids can be cultured for a long period of time and can be retrieved after culture.

However, there are some challenges such as the size and composition of the resulting spheroids (spheroid sizes can be too heterogeneous), the formation of spheroids from a small number of cells, and the setting-up of the right ratio of two different cell types in spheroids when performing co-cultures.

### 2.3. Suspension Culture

The principle of this method is to put a cell suspension inside a container and keep the cells in suspension either by agitation or by increasing the media viscosity (adding carboxymethyl cellulose) [8,25]. In the agitation-based approach, the container can either be gently stirred or rotated (Figure 1C). Agitating the cells continuously will prevent them from adhering to the container walls and will promote cell-cell interactions. There are two main apparatuses that are used in the suspension culture method: spinner flasks and bioreactors.

#### 2.3.1. Spinner Flasks

Spinner flasks consist of a container into which the cell suspension is placed and a stirring element that keeps the suspension in continuous motion. This simple method produces large yields of spheroids [25]. Changing the culture medium from the spinner flasks is relatively easy. Since the culture fluid is in constant motion, nutrients and O_2_ are transported to spheroids and their wastes can be removed [26].

However, the shear force applied to the cells in spinner flasks, resulting from the motion of the stirring bar through the cell suspension, can modify their cellular physiology [25]. Another drawback of the method is the production of a broad range of spheroid sizes, which can be an issue when they are used for drug screening assays. To tackle this, spheroids can be formed in a first step using ultra low attachment plates and can then be transferred to spinner flasks after selecting the right size of spheroids [27]. This is necessary when measuring the spheroid sizes continuously to determine the effects of the treatment on their growth.

#### 2.3.2. Bioreactors

Bioreactors are similar to spinner flasks, but instead of mixing the cell suspension with a stirring bar or a rod, the culture container is rotated. It was originally designed by NASA (National Aeronautics and Space Administration) in 1992 to culture cells and tissues during spaceflight [28]. Bioreactors are available in different sizes so that production of large amounts of spheroids is enabled. Similarly to spinner flasks, bioreactors do not allow the control of the size of spheroids, so a prior step can be included to select spheroids of the same size. Shear forces applied to cells are not as significant in bioreactors as they are in spinner flasks.

### 2.4. Scaffold-Based Models: Hydrogels

Cell-ECM interactions can modify cellular organization and cell function and response to therapy. Therefore, a 3D culture model that could recapitulate the role of the ECM in vivo would be ideal. In this context, either natural or synthetic hydrogels have been used [29]. Among the most common are naturally derived hydrogels (e.g., matrigel, collagen, alginate, and fibrin), synthetic (e.g., PEG (polyethylene glycol)) and some semi-synthetic hydrogels presenting a combination of synthetic and natural polymers (ex: hyaluronan, polypeptides) [30].

Cells can either be plated on the top of the matrix after solidification or mixed with liquid hydrogel (matrix) and then plated so that the cells get embedded within the matrix during gelation (Figure 1D). In both methods, cell culture plates are pre-coated with hydrogel. In the first method, cells are simply added on top of the hydrogel after solidification and cultured at 37 °C under agitation to allow cells to adhere to each other and form spheroids attached to the hydrogel. In the second method, cells are mixed with hydrogel and then plated on top of the pre-coated plates. Cells are incubated at 37 °C and embedded within the hydrogel when it jellifies.

Matrigel and collagen are good examples of natural scaffolds that can offer a correct cell attachment and good reorganization into 3D structures. Matrigel is a commercial ECM, which possesses Engelbreth-Holm-Swarm (EHS) mouse tumor cell-derived basement membrane proteins such as collagen IV, entactin, laminin perlecan, matrix metalloproteinase-2, and growth factors [31]. These basement membrane proteins are essential for important cellular functions like polarization, growth regulation, chemotherapeutic resistance, and adhesion [32,33]. In the ECM, collagen represents the most abundant fibrous protein. It gives tensile strength, regulates cell adhesion, and is involved in cell migration and chemotaxis [34]. Therefore, it is a good choice for studying cell behavior in vitro in a tissue-like environment. Collagen type I is mostly used in 3D cultures, but type II and III can be used as well [35,36,37,38]. Collagen offers the advantages of cell compatibility, amenability to cell adhesion without modification, and a native viscoelastic environment for cells. However, like matrigel, collagen varies from batch to batch and has a low stiffness.

Although biologically-derived scaffolds are of great interest, as mentioned above, it is difficult to have the same composition from one batch to another. Natural hydrogels also have weak mechanical properties, can degrade rapidly, and can cause immunogenic reactions [39]. The need to characterize various properties (such as mechanics, swelling, mesh size, and degradation) for each experiment can also be a drawback [30]. The variability in properties can affect the reproducibility of results and limits the use of such scaffolds for drug screening purposes.

To overcome the drawbacks of natural hydrogels, synthetic hydrogels can offer alternate options depending on the type of experiment. Using a synthetic hydrogel allows for the control of biochemical and mechanical properties, therefore improving tumor ECM mimicry. Due to their inert properties, PEG-based hydrogels need to be customized with cellular active sites in order to enable cell growth within the 3D matrices. Cell adhesion molecules, peptides, or bioactive natural polymers (collagen, fibrin) can be added to PEG-based hydrogels to enhance cellular activities [39,40]. Used in defined sizes (150 and 600 μm microwells), PEG is able to control the size of breast micro-tumors [41], reproducing size-induced micro-environmental changes such as hypoxic gradients, cellular heterogeneity, and spatial distribution of necrotic/proliferating cells, and could be useful in the study of tumor progression and antitumor drug effects.

Natural and synthetic hydrogels have their limitations to recapitulate tumor ECM, hence semi-synthetic hydrogels can be used as an alternative and do present many advantages. By providing the bioactive features of natural materials and permitting the control of chemical parameters, the semi-synthetic hydrogels can provide a controlled and defined environment. Hyaluronan, which is a major component of natural ECM, has been used in this context because it is a biocompatible, non-inflammatory, and biodegradable polymer [42]. It has a binding affinity to cell surface receptors involved in proliferation, adhesion, migration, and differentiation [43,44]. The crosslinking chemistry of hyaluronan leads to the fabrication of various hydrogels with different characteristics that are suitable for cell culture [45].

Using hydrogels is of great interest because they can have similar mechanics, composition, and structural cues as native tissues. On the other hand, when using natural hydrogels, there can be endogenous growth factors and signals that do not represent the human tumor environment, and post-culture recovery can turn out to be difficult.

### 2.5. Magnetic Levitation

Magnetic levitation was developed for the first time by Souza et al. in 2010 [46]. They used hydrogels containing gold and magnetic iron oxide (MIO) nanoparticles plus filamentous bacteriophage. In this method, cells are grown up to 80% confluence and treated with MIO-containing hydrogels and incubated overnight to allow the uptake process [47]. Treated cells are trypsinised and placed into an ultra-low attachment plate. A lid with a neodymium magnet is immediately placed on the top of the plate (Figure 1E). Spheroids start forming within a few hours at the air-liquid interface due to levitation towards the magnet. When cells aggregate to each other, they start synthesizing ECM proteins like collagen, fibronectin, and laminin [48]. Spheroids can be incubated for a few days until they reach the ideal size for study.

There are many benefits to this method: the speed of spheroid growth is high compared to the more commonly used methods; spheroids form intrinsic ECM (no need for artificial scaffold), spheroids are in the size range of mm^2^ (this size better reproduces necrotic and hypoxic area found in tumors) and, finally, they do not require any specific medium. Nonetheless, this technique presents a few drawbacks: beads are expensive and can be toxic to cells at a high concentration, and a limited number of cells can be produced.

### 2.6. Bioprinting

**Figure 2:**
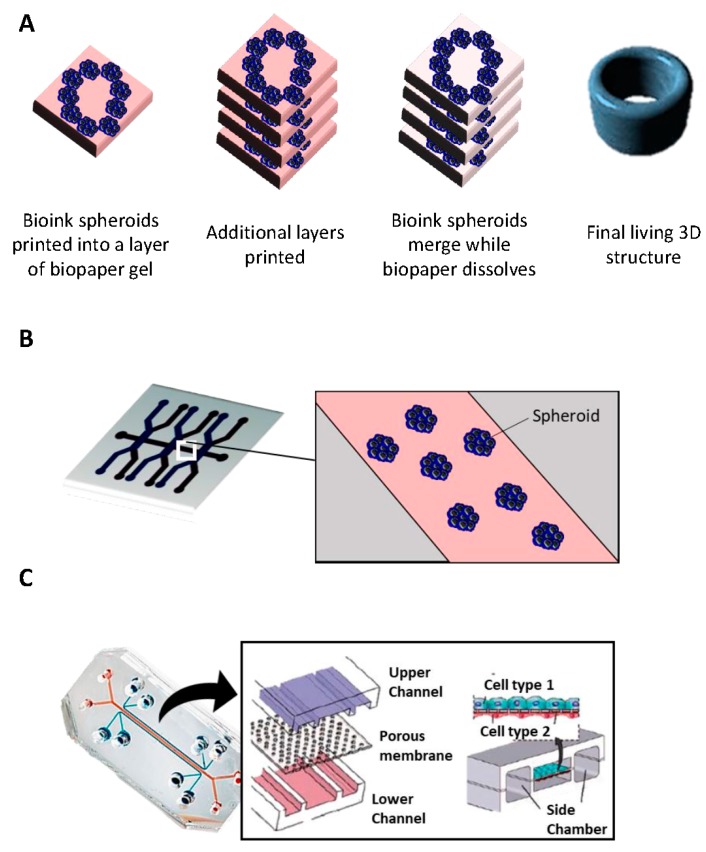
Bioprinting and Microfluidic platforms. (**A**) Biopaper gel is loaded with bioink spheroids that each contain an aggregate of a few thousand cells. More layers are subsequently added and as the biopaper gel dissolves, and the bioink spheroids slowly fuse together, leaving a final bioprinted structure. (**B**) An example of simple microfluidics system in which cancer spheroids were embedded in micro-patterned three-dimensional matrices immediately contiguous to a microchannel. (**C**) Schematic represents a two-chamber chip for the culture of two different cell types as monolayers in separate chambers that are linked through a porous membrane.

3D printing emerged thirty years ago raising promise for mass production in many different fields [49]. While current 3D models allow us to obtain different features in terms of spheroid structures, they usually have limited vascularization potential, which is critical when studying the later stage of tumor development, for example [50]. Bioprinting technology has brought technical solutions in order to increase vascularization, design a scaffold that better captures the TME heterogeneity, and, finally, provide better 3D in vitro cancer models. Specific structures are recreated by positioning precisely biological and biochemical materials and living cells layer by layer (Figure 2A). The use of medical imaging technology (MRI, CT scan, and X-ray) is therefore essential to obtain relevant information on the structure of tissues and organs [51].

Researchers use different approaches to build 3D constructs in this context: (i) biomimicry, which involves a good understanding of the microenvironment and the composition of the ECM, for example; (ii) autonomous self-assembly, which relies on the use of embryonic organ development as a guide; and (iii) mini-tissue building blocks, which are the smallest parts of tissues; they can be fabricated and later assembled to create larger constructs [52]. There are different ways to deposit and pattern biological materials: inkjet, extrusion, and laser-assisted printing [53]:

i Inkjet printing uses the assistance of a digital computer to control a narrow nozzle to generate droplets that are deposited on a scaffold in a precise and controlled manner [54]. There are four main current techniques used to create these droplets: thermal, piezoelectric, acoustic, and electrostatic.
ii Extrusion printing is another version of inkjet printing. It differs from inkjet printing by the fact that it can print on viscous materials. This is achieved through the use of an air-force pump or a mechanical screw plunger to dispense bioinks. A continuous force is applied, allowing the constant printing of cylindrical lines instead of a single bioink droplet [55].
iii Laser-assisted printing system uses a donor layer composed of a metal type energy-absorbing layer (e.g., titatium or gold) on the top and a layer of bioink solution at the bottom [56]. During the printing, a small part of the energy-absorbing layer is exposed to a focused laser beam, which generates a bubble in the bioink solution that is pushed onto a receiving substrate (where printing is achieved) [51].

Overall, by enabling the reproduction of complex in-vivo structures, bio-printing gives an opportunity to cancer biologists to replicate the TME using different bioinks (composed of ECM-like biomaterials, cytokines, and cells) [50,57]. However, when we compare the three bio-printing techniques described above, each one of them has their drawbacks, in terms of cost, resolution, cell density capacity, and cell viability. The development of new bioinks, which will be biocompatible, immunocompatible, and give the best support to the cells in their appropriate function, should be the key to improvements in this field [51].

### 2.7. Microfluidic Platforms

Microfluidics is the science that handles very small (10^−9^ to 10^−18^ L) volumes of fluids [58]. Devices contain micro-channels with dimensions of tens to hundreds of micrometers (Figure 2B,C). Microfluidics originated from four disciplines: molecular analysis, biodefense, molecular biology, and microelectronics. It finds its roots in micro-analytical methods—gas-phase chromatography (GPC), high-pressure liquid chromatography (HPLC), and capillary electrophoresis (CE)—which, in capillary format, revolutionized chemical analysis [58].

Microfluidic platforms are devices where living cells can be cultured and continuously infused in micrometer-sized chambers [59,60]. This method enables the accurate control of the cellular microenvironment, allowing a continuous release of growth factors or nutrients [58]. In the simplest system, a single microfluidic chamber contains one type of cultured cells, which possess particular characteristics of a tissue type (Figure 2B). It is also possible to study the interaction between different cell types in order to recreate interfaces between different tissues; in this aspect, micro-channels can connect to each other through porous membranes lined on opposite sides by different cell types—also known as tumor/organs-on-chip (Figure 2C) [61]. The aim is to create an environment where different cell types can interact with each other [62]. The organs-on-chip allowed us to recreate the complete complex structure and environment as skin and hair [63], lung [64], liver [65], or gut [66].

This method is convenient for high-throughput drug testing but requires specialized equipment, and post-culture recovery can be difficult. If further analysis (Elisa, western blot) is required, the low cell number can also present a drawback [67]. However, new microfluidic platforms developed recently offer solutions to this weakness. In fact, a recent two-layered microfluidic device was developed that allowed the formation, culture, and drug testing of 5000 uniform-sized tumor spheroids with different culture chamber geometries (200 × 200 μm^2^ and 300 × 300 μm^2^) [68]. In addition, cutting-edge western blot tools (“Single-cell western blotting”) or single-cell transcriptomic can counterbalance the low cell number problem.

## 3. Tumor-Microenvironment Interactions: 2D vs. 3D

For a long time, tumor initiation, progression, and metastasis were seen to be merely due to changes in the neoplastic cell population and the fact that the adjacent non-neoplastic tissues were regarded as bystanders. The importance of the TME emerged from the observation that histopathological sequence changes were found at the interface between putative tumor cells and the surrounding non-neoplastic tissues during carcinogenesis. It is now known that the TME is a specialized entity: dynamic, interactive, and constantly changing [69]. The TME comprises different cells that are collectively important for normal tissue homeostasis as well as tumor progression, migration, and resistance to chemotherapy. In order to better understand such mechanisms and develop the appropriate blocking strategies, one needs to use appropriate complex models of tumor and microenvironment culture.

Although 2D cultures are well accepted and have pointedly helped our understanding of cell behavior, it is now proven that 2D systems result in cell bioactivities that are different from the in vivo response. For instance, some important cancer cell features (such as morphology genetic profile or tumoral heterogeneity) are not correctly modeled in 2D cultures (Table 1). 3D cell culture platforms help to circumvent these limitations by preserving the original shape, polarization, genetic profile, and heterogeneity of cancer and stromal cells. Although these might suggest that 3D culture should always be used, the lack of a universal 3D model and the simplicity of 2D culture are the main reasons why the platform of choice is not often dictated by the specific process of interest. Moreover, the assessment of drug efficacy or growth required the precise measurement of cell viability or metabolism. Depending on different 3D culture conditions/setups, it may not be easy to perform regular MTT or live/dead staining. In addition, in co-culture situation, how to distinguish the growth/death of two cell types can be trickier. Therefore, complex quantification methods as flow cytometry or single cell western blot have to be used.

Here, we provide an overview of the different 3D culture methods chosen in the literature while studying tumor-microenvironment interactions in the context of cancer cell proliferation, migration, and resistance to treatment, and discuss the differences between 2D and 3D.

### 3.1. Proliferation

**Figure 3:**
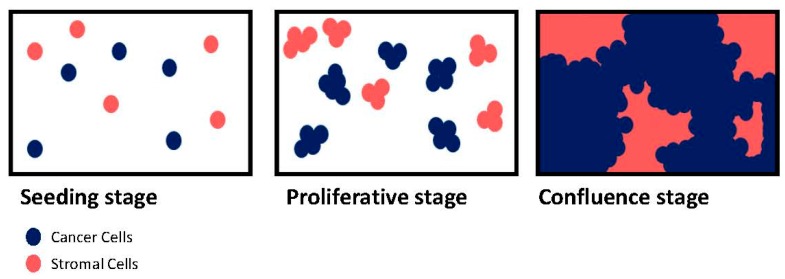
Cancer and stromal cells co-culture in 2D. Schematic illustrates cell behavior at different stages (seeding, proliferative phase, and confluence) of a 2D culture model. Stromal cells (in red) and cancer cells (in blue) have the same access to the media growth factors. Cells with contact inhibition (stromal cells) slow down their division while the space availability decreases until complete stop. Cells with no contact inhibition (cancer cells) keep proliferating and start to grow on the top of other cells.

One of the main characteristics of cancer cells is their high speed of growth and their loss of contact inhibition [70]. Contact inhibition is a regulatory mechanism that allows mammalian cells to grow only into a monolayer [71]. If the available space is large, cells will divide rapidly until they occupy the full surface. At this point, normal cells will initiate cell cycle arrest to stop proliferation [72]. By contrast, cancer cells proliferate indefinitely and display no contact inhibition. Consequently, in a classical 2D culture system, once the whole culture dish is occupied, the cells keep dividing, piling up into mounds [73]. This is a significant problem during co-culture between cancer and TME cells (Figure 3). In fact, TME cells are normal cells subject to contact inhibition while cancer cells are not. Therefore, the seeding density as well as ratio between two cell types will have to be considered during the design of the experiment. During co-culture, TME cells slow down their proliferation when confluence is imminent but cancer cells do not.

For instance, MCF-7 (Michigan Cancer Foundation-7), a breast cancer cell line, cultured either in 2D or 3D on a collagen scaffold, displayed differences regarding their morphology and proliferation [74]. When seeded in 2D, they demonstrated sheet-like, trigonal, or polygonal morphologies, whereas when in 3D collagen scaffolds, they showed round, shuttle shape-like and spread-out appearances. While MCF-7 grew at the same speed in 2D and 3D during the first five days, MCF-7 cells cultured in 2D stopped growing at day 7, while those in 3D continued expanding even after 13 days. This can be explained by the fact that the cells growing on the top of each other start to die. With another set-up, Cavo et al. studied the effect of substrate elasticity on MCF-7 [75]. In 3D conditions within mechanically tuned alginate hydrogel-coated dishes, cells formed round and nicely organized spheroids, similar to what could be found in vivo, whereas in the 2D condition, they showed a flat shape. The authors demonstrated a direct link between cell viability and substrate elasticity. When the substrate elasticity went up, the number of MCF7 would decrease. This study highlighted how 2D cultures are not representative of a real in vivo set-up.

While simple cultures are already biased by a 2D culture system, co-cultures with TME cells increase the challenge. In our laboratory, we co-cultured breast cancer cells (BCC) with EC in 2D and 3D models of organized angiospheres on ultra-low attachment plates [76]. The EC were able to enhance the proliferation of the BCC in both settings. However, BCC proliferation was more pronounced in 3D models, and the experiments could be performed over a longer period. In another study, Majeti et al. developed a 3D co-culture model, using 96 well plates coated with poly-2-hydroxyethyl methacrylate, where pancreatic, breast, and lung tumor cells were grown in co-culture with human fibroblasts [77]. They revealed that 3D co-culture had a different impact on cell survival compared to 2D co-culture. Additionally, when co-cultured in a 3D condition in presence of fibroblasts, cancer cells had a higher proliferation rate.

One of the co-culture challenges is to keep a ratio between the two cell types that reflects in vivo setting, considering the proliferation rate of each cell type and their properties when cultured in vitro. In their study, Eder et al. used the hanging drop method on prostate cancer cells co-cultured with cancer-associated fibroblasts [78]. They showed an increased number of cancer cells compared to fibroblasts within the spheroids, which reflects what is observed in vivo. Using this method, the authors were able to avoid the artefact of a non-representative ratio between different cell types usually observed in 2D models.

3D culture methods recreate a more complex environment than simple 2D co-cultures, allowing a better reproduction of the in vivo environment. For instance, Jaganathan et al. used the magnetic levitation method, allowing the formation of spheroids without the use of a scaffold [79]. Unlike 3D methods using a scaffold, cells here could form heterogeneous aggregates with each other and produce a tumor-like hypoxia and necrotic regions similar to in vivo tissues. They compared the phenotype of their model to the in vivo tumor and demonstrated the presence of similar ECM proteins. Their method was advantageous due to the ability to form large-sized spheroids within 24 h, control the tumor cell composition and density, and mimic the TME. Recently, Brancato et al. confirmed the 3D model consistency with an in vivo setting, using biodegradable microcarriers (made of gelatin) in a spinner flask bioreactor [80]. In their system, pancreatic cancer cells were co-cultured with cancer-associated fibroblast, resulting in the formation of human pancreatic ductal adenocarcinoma microtissues. Cancer cells were shown to be the predominant proportion of proliferating cells, which is consistent with other 3D co-cultured systems. The expression modulation analysis of ECM genes and proteins showed that when fibroblasts were cultured with pancreatic cancer cells, they converted into myofibroblasts and expressed desmosplastic markers. This new platform demonstrated how important the stroma-cancer cross talk is for both cell types. Santo et al. demonstrated the robustness and reliability of stirred-tank cultures for the generation of 3D cancer models [81]. They used a panel of tumor cells to obtain a large number of spheroids by tuning hydrodynamic parameters. They were able to produce spheroids that present features of native tumors such as morphology, proliferation, and hypoxia gradients in a cell-line dependent mode. They successfully produced heterotypic 3D cancer models by co-culturing tumor cells and fibroblasts in absence or presence of an additional alginate matrix that was preserving high cell viability. More recently, Chung et al. used a new model that recapitulates the interactions that exist between the different constituents of the TME during tumorigenesis [82]. In this model, they mimic the interactions between tumor and stromal cells. By using a microfluidic platform, they were able to induce simultaneous angiogenesis and lymphangiogenesis, thus demonstrating how they affected cancer cell proliferation. Such a platform is a good tool to study the complex physiopathology of the TME, as illustrated by a study using a two-layer microfluidic system to culture metastatic prostate cancer cells, osteoblasts, and endothelial cells [83]. This culture system allowed the metastatic prostate cancer cells to reduce their growth without losing viability, leading to a proliferation rate closer to the in vivo behavior of malignant cancer cells within the bone metastatic prostate cancer microenvironment.

### 3.2. Migration and Invasion

Cell migration is a normal process in all multicellular organisms; it is essential for normal development and has a crucial role in different processes such as wound healing and immune responses. Abercrombie and Heaysman, who studied how a cell behavior could be influenced by other cells, were the first ones to observe that when a cell was in proximity to other cells, not only would it reduce its motility, but it would also change its direction [84]. They stated that a cell would always preferentially adhere to a substrate rather than to its neighboring cells. They named this restriction phenomenon: CIL (contact inhibition of locomotion) [73,85,86]. The loss of a normal CIL in many cancer cells is the reason behind contacts with non-cancer cells [87]. This new migratory behavior could promote cancer cell invasion that could be enhanced through interactions with stromal cells. Interestingly, cancer cells still possess CIL when they encounter other cancer cells, which means that their failure in displaying normal CIL when contacting non-malignant cells could come from defective signaling rather than a general lack of contact inhibition mechanisms [88,89]. This phenomenon plays a major role during the metastasis process. In fact, during malignant progression, tumor cells earn the ability to invade neighboring tissues and propagate in distant organs through a phenomenon called EMT (epithelial to mesenchymal transition) [90]. During EMT, cancer cells gain a mesenchymal phenotype that allow them to go beyond the basement membrane and degrade the ECM with metalloproteinases, to finally reach the bloodstream. Cell migration, in this context, is the result of critical events: polarity changes, loss of cell-cell or cell-ECM contacts, and the formation of actin rich protrusions (ex: filopodia and lamellipodia) due to a chemoattractant gradient and migratory stimuli [91,92].

Most of the studies and discoveries on cell migration were made using 2D models [93]. 2D models induce a flatness of the cell-motile appendages. Migration in 2D is governed by classic polarized signaling and mechanical patterns that are often not crucial for effective migration in 3D [94]. Moreover, there are more modes of motility in 3D that are very complex and take into consideration not only stiffness but also the rheology and geometry of the ECM. For instance, our team worked on SDF-1α and CXCR4 roles in the metastasis process of BCC in monoculture and co-culture with stromal cells [95]. We concluded that SDF-1α concentration gradient modulates migration and adhesion of BCC, by controlling the expression and activation of Rho GTPases (guanosine triphosphatases) in 2D culture. The principal drawback of our study is that we limited our work to a 2D model. Different concentrations of SDF-1α might not have the same effect in a 3D model.

Besides, cells cultured in 2D or 3D often do not express the same cell surface proteins. Loessner et al. showed that the mRNA expression of the cell surface receptors α3/α5/β1 integrins and the protease MMP9 were increased in ovarian cancer cells in 3D culture compared to cells in 2D culture [96]. In 3D culture (made on Matrigel), CXCR4 expression was altered in prostate cancer cell lines, which regulate the metastatic process of prostate cancer cells [97].

The complexity increases when one wants to add another cell type to the culture. As stated earlier, the TME is very important to the migration process of cancer cells. Therefore, it is critical to have proper 3D models of co-culture that allow the study of the effect of stromal cells in cancer cell migration. As early as 2005, Winters and collaborators were already proposing the use of 3D multicellular spheres to study tumor biology to better estimate the interactions encountered by cells in vivo [98]. They showed that intercellular cohesion within spheroids was predictive of the invasion potential in vivo. In our laboratory, we showed that BCC co-cultured in spheroids with activated EC, increased BCC tumorigenicity, stemness, and invasiveness [76]. Simultaneously, we demonstrated that EC also benefit from this cross-talk by gaining mesenchymal markers [99]. Both phenotypic changes in endothelial and BCC in the spheroid model lead to an increase of the BCC invasion potential in vitro and in vivo. More recently, Chen et al. used a single cell-based microfluidic approach to co-culture single breast cancer cells and primary cancer associated fibroblasts on-chip for 14 days to monitor sphere formation and growth and perform single cell transcriptome analysis [100]. The authors demonstrated that tumor-stromal interactions induced expression changes in genes associated with proliferation, apoptotic suppression, tumorigenicity, and EMT in BCC co-cultures with the primary cancer associated fibroblasts.

The multicellular spheroid model is of great interest as it has been shown that spheroid structures could be isolated from ascites fluids. Burleson et al. showed that spheroids recovered from the ascites fluid of patients with stage III or stage IV ovarian carcinoma could adhere directly to ECM through integrins, and then degrade type I collagen to invade the peritoneum [101]. More recently, TGFβ signaling has been shown to regulate EMT in spheroids derived from ovarian cancer ascites leading to the promotion of malignant properties of these structures [102]. Many studies state that spheroids found in ovarian cancer patient ascites could be responsible for peritoneal metastasis highlighting once more the importance of 3D spheroid model in migration/invasion studies [103,104,105].

3D culture systems have also enabled the study of more complicated phenomena. For instance, in order to study breast cancer metastasis following extravasation to bone, Bersini et al. developed a tri-culture system using a microfluidic 3D model [106]. Using human osteo-differentiated bone marrow-derived mesenchymal cells and EC, they were able to build an osteo-cell conditioned microenvironment. In this model, highly metastatic human BCC (MDA-MB-231) crossed the endothelial monolayer and reached the collagen-embedded osteo-differentiated human bone marrow mesenchymal stem cells. Extravasation was observed 24 h after cancer cells were introduced to the endothelial channel and was significantly higher in the osteo-cell conditioned microenvironment compared to collagen gel-only matrices. They also measured a higher migration distance of the cancer cells when they were in the osteo-cell conditioned microenvironment compared to collagen gel-only matrices. CXCR2 (breast cancer cell receptor) and CXCL5 (bone-secreted chemokine) were shown to play a major role in the extravasation process as well. Wang et al. tri-cultured MCF10A cells with fibroblasts and human adipose-derived stem cells in 3D and demonstrated that MCF10A expressed higher levels of α-casein mRNA, an indicator for functional differentiation of mammary epithelial cells, compared to MCF10A in monoculture or co-culture with either cell type [107].

### 3.3. Resistance to Treatment

Despite tremendous improvement in diagnosis and therapy, cancer mortality remains high [108]. While prognosis of early stage cancers is quite good after treatment, advanced stage cancers are often not curable and patients usually die from metastatic burden despite several lines of chemotherapy. Treating cancer metastasis has been challenging due to the complexity and heterogeneity of tumors, as well as chemotherapy resistance. To overcome chemotherapy resistance, hundreds of drugs have been developed and tested in clinical trials for cancer treatment over the past sixty years [109]. The most common and oldest type of drug used in cancer treatments is cytotoxic agents. They are non-specific intracellular poisons that usually target highly dividing cells, a classical feature of cancer cells. Recently, with the emergence of high-throughput sequencing, targeted therapies against specific molecular or genetic targets have been developed.

#### 3.3.1. Cytotoxic Agents

Despite the development of potent cytotoxic drugs against many cancer types at different sites, cancer cells will develop drug resistance driven by tumor heterogeneity, drug inactivation, apoptosis evasion, enhanced DNA repair, increased drug efflux, and EMT [110]. For a long time, the relative failure of anti-tumoral treatment was attributed merely to the genetic of cancer cells and cellular processes occurring within cancer cells. However, over the last decade attention has shifted toward the TME role in development of chemoresistance [111]. In a solid tumor, drugs need to access every cancer cell to be effective; thus, the diversity within the TME in terms of stromal cells, ECM, oxygen availability, and environment acidity have become important players in cancer cell response to drugs.

**Figure 4:**
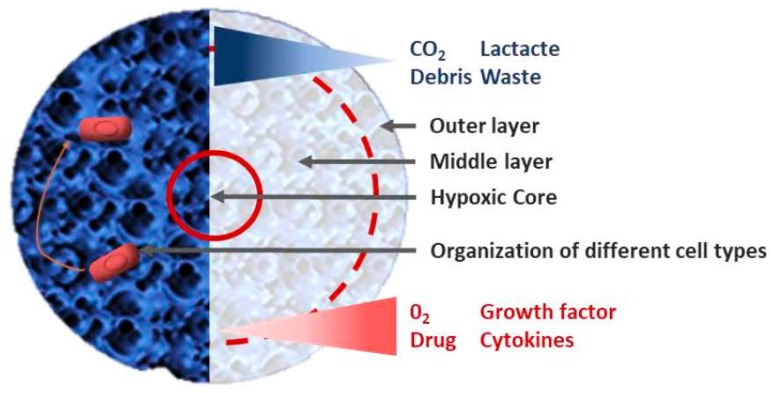
Spheroid organization Tumor spheroids are organized in three different layers with a dying core, a quiescent intermediate layer and a proliferating rim. Stromal cells (in red) are organized in a different way inside the spheroid depending of the context.

The communication between cancer cells and their TME is bi-directional and much more complex than first supposed. Both tumor and stromal cells are exposed to different concentrations of drugs over the course of treatment and they develop a cooperative relationship that seems to benefit cancer cells. Therefore, it is now primordial to develop cell-based assays that take into account the component of the TME to evaluate the possible efficiency of a new compound in cancer drug discovery. In 2D, stromal and cancer cells are exposed to the same drug concentration, oxygen amount, or environment acidity. Moreover, the cross talk between adjacent cells are limited to spatial and physical aspects of 2D cultures. In 3D model, the architecture is more complex; spheroids are commonly organized in 3 layers: (i) an outer layer with a high proliferation rate, (ii) a middle layer with senescent cells, and (iii) a hypoxic core with necrotic cells (Figure 4) [112]. The level of oxygen and nutrients is lower in the core due to cell density and ECM increase, which reflects the conditions that are observed in vivo in solid tumors, which can have up to 60% of their volume in a chronic hypoxia state [113]. The constant hypoxia in the core leads to glycolysis and synthesis of CO_2_, pyruvate, and lactate resulting in an acidification of the medium. Cells have to adapt to this hostile environment to grow. Therefore, some cytotoxic agents such as doxorubicin, 5-FU or cisplatin, which work with oxygen, are less effective in 3D models [114,115], while other drugs like tirapazamin, which are more effective on hypoxic cells, have a greater effect on spheroids and their hypoxic core [116,117]. A good illustration would be the study of Tung et al. [16]. They used a 384 well format hanging drop culture plate to test two different drugs: 5-fluorouracil (5-FU, a cell proliferation inhibitor) and tirapazamine (TPZ, a hypoxia-activated cytotoxin) on three different cell types: African green monkey kidney fibroblast cell (COS7), murine embryonic stem (mES) cell (ES-D3), and human epithelial carcinoma cell that stably express mesothelin (A431.H9). In their study, the authors showed that H9 cells were more resistant to 5-FU when they were treated in 3D cultures compared to 2D cultures. On the other hand, the hypoxia-activated drug TPZ was more effective against 3D cultures. They also showed that taxanes have a loss of efficiency on spheroids, which is interesting because this family of chemotherapy is offered in the first-line treatment (either as neo-adjuvant or adjuvant) in ovarian cancer (5% viability in 2D and 75% in 3D with the same treatment, suggesting a chemoresistance of the spheroids). Oxygen is also an essential element for photodynamic therapy (PDT), which is an alternative to traditional cancer chemotherapy generating local oxidative stress to kill cancer cells using photosensitizer, oxygen, and light [118]. Chen et al. used a microfluidic sphere formation platform to study tumor associated fibroblasts in resistance to traditional chemotherapy agents and PDT in breast cancer cells [119]. They concluded that tumor associated fibroblasts were not able to induce resistance while using PDT, suggesting that PTD could be less affected by resistance induced by cancer associated cells.

Many authors have compared drug efficacy of cytotoxic agents between 2D and 3D [120,121,122]. For instance, Huber et al. cultivated the non-small lung cancer cell line Colo699 in monolayer (2D) on plastic plates for 5 days and as micro-tissues (3D) using HDP for 5 and 10 days [123]. Cells and micro-tissues were treated with different drugs: afatinib (10–80 μM), cisplatin (100–800 μM), or vinorelbine (25–200 μM) for 24 or 48 h. The authors showed significant differences in drug efficacy between the 3D and 2D cultures. 3D cultures displayed resistance to cisplatin and vinorelbine but were sensitive to afatinib, demonstrating that the 3D model was a relevant in vitro system closer to the in vivo system. Differences between 2D and 3D also exist in presence of stromal cells. Houshmand et al. recreated a leukemic bone marrow niche in order to study chemoresistance [124]. They co-cultured TF-1 cells and bone marrow mesenchymal stem cells in 2D and 3D, and treated them with azacitidine and cytarabine. Cytoxicity assays showed a higher resistance in spheroids compared to cells cultured in 2D due to Bcl-2 overexpression. In a more complex model of microfluidic 3D hepatocyte chip, Toh et al. engineered a 3D microenvironment to predict in vivo drug hepatotoxicity [125]. They evaluated the IC_50_ values of 5 model drugs and demonstrated that they were consistent with their in vivo LD_50_ values.

Nevertheless, the major drawback of the 3D model while studying chemoresistance toward cytotoxic agents is the selection of cancer stem cells (CSC) population. In fact, culturing cancer cells in 3D leads to the acquirement of a stem-cell like phenotype with increased expression of nestin, CD44, RHAMM, and CD133 [126,127]. CSC are more prompt to grow in a 3D anchorage-independent culture setup [128]. Primary or secondary sphere formation is commonly used to enrich and quantify the stem cell population [129]. The prominent characteristic of CSC is their chemoresistance [130,131,132]. Indeed, CSC population is known to overexpress ATP-binding cassettes (ABC) transporters and therefore displays a resistance to drugs and toxins [133,134]. ABC transporters are one of the main actors of the multidrug resistance in cancer cells. They are small efflux pumps that can expulse most of the cytotoxic agents used in chemotherapy. Thus, it is more likely that cancer cells will be more resistant to chemotherapy in a 3D setting than in 2D due to the increase of the CSC population. In ovarian cancer, CD44^+^CD117^+^ spheroids displayed a higher chemoresistance and were able to initiate and propagate tumors in mice model [130]. Similarly, Luo et al. described that CD117^+^ cells isolated from xenografts were resistant to chemotherapy and exhibited phenotypic features of CSC such as asymmetric division and serial transplantation [135]. Gao et al. described that CD24^+^ population expressed increased levels of stem cells genes such as Nestin, β-catenin, Bmi-1, Oct4, Oct3/4, Notch1, and Notch4 compared to CD24^−^ and were able to initiate tumor formation and resist to cytotoxic treatment [136]. Recently, Wang et al. synthesized a 3D porous scaffold composed of chitosan and hyaluronic acid, and showed that when glioblastoma cells GBM6 were cultured in such condition, they formed spheroids, which displayed a closer cellular morphology to CSC [137]. These cells were more resistant to alkylating agent-based chemotherapies (used in postoperative treatment of glioblastoma). For example, estimated lethal dose, 50% (LD_50_) for the methylating agent TMZ of cells cultured in 3D, was three times higher than cells cultured in 2D condition.

Another limitation of the 3D model is the ability of cells to form tight spheres. In fact, depending on the cell type or line, the sphere can be more or less compact. For instance, for some cell types, such as MDA-MB-231 breast cancer cells, it is difficult to aggregate tight spheres as compared to MCF7 [138]. The compactness of a sphere may affect the estimation of cell number and interfere with drug efficacy.

#### 3.3.2. Targeted Therapy

To overcome resistance to cytotoxic agents, targeted therapies have been developed [139]. Targeted therapies are usually small-molecules, monoclonal antibodies, and immunotoxins, which aim to prevent the action of certain genes or proteins specifically expressed on cancer cells [140,141,142]. The advantage of such treatment, in theory, relies on the fact that, unlike cytotoxic drugs, which give rise to many off-target side effects to normal tissues, the release of drug to cancer cells acts directly on specific molecular targets (overexpressed protein such as her2neu or EGFR and ALK) that are associated with cancer. Therefore, targeted therapies should be less harmful to normal tissues [143,144]. Since the end of the 90’s, more than forty targeted therapies have been approved in cancer treatment [139]. Unfortunately, the response rate is often deceptive, with only a slight effect on prognosis and no major increase in the overall outcome [145]. This is mainly due to an intrinsic resistance to targeted therapies by the tumor cells (also known as primary or de novo resistance) or acquired resistance over the time of treatment (known as acquired resistance) [146]. There are three main reasons why resistance to targeted therapies happens: (i) alteration of a driver oncogene: the oncogene is mutated so it displays a different form and is not recognized by the inhibitor, which results in proliferation and cell survival; (ii) activation of a critical signaling pathway in a parallel or downstream way: the inhibitor can block the action of the oncogene but the upregulation of a distinct receptor continues the signaling or the mutation of a protein down the signaling pathway (below the targeted protein) reactivates the pathway; and (iii) activation of pro-survival signaling through an alternative signaling pathway, which can block the action of inhibitor-mediated apoptosis [147]. Interactions with stromal cells of the TME play a major role in the development of these mechanisms of resistance [148]. Apparition of 3D models led to a better representation of a tumor, giving a more realistic drug response equivalent to clinical observations compared to 2D models. For instance, 3D cultures better reflect HER2 intracellular signaling pathway compared to 2D cultures [149]. Injection of trastuzumab (a monoclonal antibody used on HER2-positive breast cancer patients) into 3D spheroids was more effective compared to 2D conditions (proliferation reduction of 48% for 3D and 16% in 2D), in concordance with clinical data. Yang et al. showed that lung cancer cells are more resistant to Bortezomib (proteasome inhibitor) in association with TRAIL when they are cultured in 3D [150]. Interestingly, they observed a modification of Bcl-2 family ratio that is responsible for the acquired multicellular resistance.

Among monoclonal antibodies used in targeted therapy, the anti-angiogenic drugs play an important part. They aim to inhibit cancer angiogenesis (formation of new blood vessels from preexisting vessels), a major event in cancer progression allowing oxygen and nutrients delivery to cancer cells in order to promote tumor proliferation and metastatic spread [151]. Bevacizumab, a humanized monoclonal antibody blocking the binding of vascular endothelial growth factor (VEGF) ligands to their receptors, was the first anti-VEGF agent approved by the U.S. Food and Drug Administration for cancer patients [152]. Targeting its pathway has been very promising, but at certain cancer sites, studies demonstrated its lack of effectiveness due to intrinsic resistance mechanisms that take place and lead to drug insensitivity [153,154]. For instance, our team demonstrated that cancer cells were able to protect EC from bevacizumab in 2D and 3D models [153]. We demonstrated that in 3D, spheres with ovarian cancer cells and EC resisted to bevacizumab treatment and were even able to proliferate.

VEGF is not the only proangiogenic factors playing a role in angiogenesis; other factors such as FGF (fibroblast growth factor) and PDGF (platelet-derived growth factor) are in the top list of these factors with distinct roles [155]. Most of the proangiogenic factors are part of the Tyrosine Kinase family; therefore, many tyrosine kinase inhibitors emerged such as sorafenib, sunitinib, or axitinib. In their study, Chiew et al. used a 3D platform (non-adhesive round bottom 96-well plates pre-coated with 1% Pluronic-F127) to study the interaction between EC and HepG2 hepatocellular carcinoma cells [156]. In their model, tumor cells were able to interact with EC creating spheroids with a hypoxic core that is physically closer to real tumors. EC were able to form tubule networks inside the 3D structure. They evaluated the effect of sorafenib, sunitinib, and axitinib by detecting apoptosis in EC and demonstrated that their tumor spheroid model had a hypoxic core leading to a more realistic penetration gradient of drugs compared to 2D models. Additionally, McIntyre et al. demonstrated that CAIX, a key hypoxia regulated gene, was upregulated in 3D culture and in vivo, but not in 2D [157]. Moreover, CAIX expression was associated with a diminished response to bevacizumab in colon cancer and glioblastoma. They demonstrated that combining inhibition of CAIX and anti-angiogenic treatment reduced the tumor growth. This study highlighted that, as with cytotoxic drugs, hypoxia is also an important factor to take into account when assessing antiangiogenic efficiency.

## 4. Conclusions

2D cell culture has been an essential tool for over a century to help researchers understand the mechanisms that underlie cell behavior in vivo. In cancer research, well-established and characterized 2D cancer cell culture has massively contributed to the understanding of carcinogenesis from cell proliferation and migration to drug discovery. Yet, cancer remains a complex disease that is still not fully understood, especially due to its close interactions with its surrounding cells. While the tumor-TME interactions have become the focus of many laboratories, the way to study them is not always clear.

2D cultures generally fail to translate accurately the natural in vivo setting. When cells are cultured in 2D, they grow as monolayers, which lead to polarized cell adhesion and two-dimensional contact with neighboring cells. This physical characteristic allows them to receive a homogeneous amount of nutrients and growth factors from the media, resulting in abnormal cell spreading, an unrealistic distribution of cell surface receptors, and selection for specific cell sub-populations best adapted to 2D in vitro growth. These limitations have encouraged the emergence of many 3D methods to better translate the complex pathophysiological features of the TME in vitro. 3D models can mimic a tumor process through its heterogeneity, giving a better reflection of the real tumor setting and a more natural response to different soluble factors present in the TME. The important diversity of existing 3D methods urges scientists to find the most appropriate one to study particular cellular and physico-chemical aspects of the TME.

An issue that is often faced when using standard 3D models is that they either create false interactions between the cells and their matrix or they have an over-simplistic model of tumor. For instance, it is possible to design an experiment using a hydrogel where cells can be in an environment that will closely mimic in vivo interactions; however, a false background can also be created. To circumvent this issue, one can use a technique where spheroids are created without any interaction with an external scaffold, but it would again give a limited view of the full picture. Moreover, as mentioned previously, culturing cells in spheroids can also induce a selection of certain populations like the CSC. This unrealistic selection will lead to an increase of chemoresistance toward many cytotoxic compounds. This demonstrates that like 2D methods, 3D methods are also able to create their own culture artefact or population selection, which is inherent to in vitro culture.

Among 3D techniques, the microfluidic platform is one of the most promising methods, because it exceeds the standard 3D models in the re-transcription of a tissue-like structure. The system reproduces cell-cell interfaces, growth factor gradients, and mechanical properties of the TME with a high level of accuracy compared to other methods. However, this technique can be expensive, and requires specialized equipment.

Cell culture is an indispensable tool to help uncover fundamental cancer processes and new therapeutics. Although flat, 2D cell culture has dominated in vitro study for a long time, it is now clear that research has shifted into the direction of culture using 3D structures, with more realistic biochemical and biomechanical microenvironments. 2D cell culture methods can still be accurate for numerous bioactivities, and new developments in substrate design will bring new capabilities for this platform. Overall, 3D systems are likely to offer an increasingly attractive substitute for 2D cell culture as advances in the field produce a wider variety of methods. A sensible approach for now would be to combine the best-adapted 3D method with a classical 2D culture to improve the selection of new therapeutic targets before pre-clinical assessment of chemotherapies.

## Figures and Tables

**Table 1:** Main differences between 2D and 3D when studying tumor microenvironment interactions.

	2D	3D
Morphology	Shape changed Polarization lost	Real shape Polarization conserved
Genetic profile	Cellular adhesion, proliferation and survival genes are modified compared to in vivo	Better representation of growth factors, pro-angiogenic and adhesion molecule genes
Cell differentiation Morphogenesis	Non spontaneous	Could be spontaneous via cellular contact or soluble factors
Angiogenesis	Only observational	Can be functional
Tumoral heterogeneity	Basic	Better approximation via the proliferation gradient, drug penetration and difference in mobility
Mathematical model	Possible	Better geometry, better link between structure and function
Reproducibility	Short term only	Controversial
Cost	Affordable	More expensive especially with some techniques
Multicellular study	Better when studying the immune response	Good in co-culture, but complicated with more than two cell types
